# Superficial Urothelial Cancer in the Prostatic Urethra

**DOI:** 10.1100/tsw.2006.402

**Published:** 2006-02-28

**Authors:** Ziya Kirkali, A. Erdem Canda

**Affiliations:** Dokuz Eylul University School of Medicine Department of Urology, Izmir, Turkey

**Keywords:** bladder cancer, superficial bladder tumors, prostatic urethral tumor, transitional cell carcinoma

## Abstract

Transitional cell carcinoma (TCC) is a multifocal disease of the urinary tract that can also involve the prostatic urethra (PU). The exact incidence of superficial involvement of the PU in patients with bladder TCC is not well known. Bladder TCC may involve the prostate in 12—40% of the patients and the degree of involvement can include urethral mucosa, ducts, acini, and stroma of the gland, which has been shown to affect the outcome. Risk factors for superficial urothelial cancer in the PU are high-grade, multifocal bladder TCC and presence of carcinoma *in situ* (CIS) in the bladder. While visible tumors are easy to detect and resect, controversy still exists regarding the optimal technique to identify prostatic involvement by TCC. Prostatic urethral sampling by a transurethral resection biopsy or a cold-cup biopsy, particularly in the high-risk group of bladder cancer patients, has been recommended for detecting prostatic urethral involvement. Management of superficial prostatic involvement by TCC is also unclear. Currently, there is increasing recognition of the value of conservative treatment options with intravesical agents when there is superficial involvement of the PU. Particularly, intravesical bacillus Calmette-Guèrin (BCG) seems to be an effective treatment alternative in the management of superficial involvement of the PU by TCC. Close follow-up by cystoscopy and PU biopsy at 3-month intervals, particularly in intermediate and high-risk patients who respond to intravesical therapy and in whom cystectomy is appropriate, is recommended in order to detect persistent tumor, recurrences, or progression.

## INTRODUCTION

Transitional cell carcinoma (TCC) is a multifocal disease that can develop anywhere in the entire urinary tract, including the prostatic urethra (PU). The mucosa lining the PU and the prostatic ducts have the same transitional cell lining as the bladder; therefore, TCC can originate from or invade the prostate. The degree of prostatic involvement can include urethral mucosa, ducts, acini, and stroma of the gland, which has been shown to affect the outcome[1]. The exact incidence of superficial involvement of the PU in patients with bladder TCC is not well known and its management is still unclear. In this article, a brief update is presented on superficial urothelial cancer in the PU.

## DEVELOPMENT

Superficial involvement of the PU by TCC is not clearly understood and it appears that there are three possible ways in which TCC can develop in the PU.

1. **Extension of the bladder TCC into the PU:** Previously developed bladder TCC may extend from the bladder into the PU.
2. **Implantation of the TCC cells shed from the bladder TCC on the PU:** TCC cells shed from the primary tumor in the bladder can implant in the PU. Although it was suggested that free-floating tumor cells from the bladder tumors may implant at the traumatized sites particularly by instrumentation[2], it has recently been shown in a prospective study that simultaneous transurethral resection (TUR) of the bladder tumor and TUR of the prostate do not negatively affect tumor recurrence at the bladder neck and PU[3].
3. **The impact of carcinogens on the PU:** Because the mucosa lining the PU and the prostatic ducts have the same transitional cell lining as in the bladder, carcinogens playing a role in the development of bladder TCC may cause the same process in the PU, leading to the development of primary prostatic TCC.

## INCIDENCE

Primary TCC of the prostatic urothelium is rare, occurring in 1–4% of patients[4], but development of TCC in the PU during follow-up of bladder cancer patients is more common and has been reported in 12–40% of patients[5,6]. The exact incidence of superficial involvement of the PU in patients with bladder TCC is not well known and since PU biopsies have not been done routinely, the incidence of PU involvement might be higher than expected[7]. Prostatic urethral involvement was detected to be 7–43% in the cystoprostatectomy specimens of the patients treated for bladder cancer[8,9,10,11,12]. Mungan et al.[13] reported the incidence of superficial PU involvement as 6.2% in patients with superficial TCC of the bladder. The development of TCC in all celular layers of the prostatic urethral mucosa is considered as carcinoma *in situ* (CIS) of the PU and its incidence has been reported to be 8.6–36%[14].

Herr and Donat[15] evaluated a series of 186 male, superficial bladder TCC patients with a follow-up of 15 years. Noninvasive prostatic tumor (prostatic urethral and ductal) was detected in 45 patients (24%) and stromal invasion was detected in 27 patients (15%) after a median follow-up of 28 months with survival rates of 82 and 48%, respectively. They concluded that relapse of superficial bladder TCC in the prostate may portend tumor invasion in the bladder, stromal invasion in the prostate, and has a significant impact on survival.

## RISK FACTORS

Multiple and recurrent superficial TCCs of the bladder recur more often than solitary and primary tumors, high-grade tumors more frequently than low-grade tumors, and $pT_1$ more frequently than $pT_a$ neoplasms[16,17]. Prostatic urethral TCC has been detected to be more common in bladder cancer patients with high-grade, multifocal tumors with CIS; therefore, routine sampling of the PU by TUR biopsies have previously been recommended in patients at high risk[18] (Fig. 1).

Herr[19] reported on 307 patients with multiple recurrent papillary TCCs and CIS of the bladder who were treated with TUR and intravesical BCG with a median follow-up of 12 years. Tumors were detected in the PU or ducts in 61 out of 251 men (24%) and tumors in the upper urinary tract (UUT) in 78 out of 307 patients (25%). It was concluded that tumor relapse risk is increased in the extravesical sites such as PU and UUT in patients with high-risk superficial bladder tumors. Extravesical tumors were detected during investigation of a positive urine cytology after no tumor was found in the bladder.

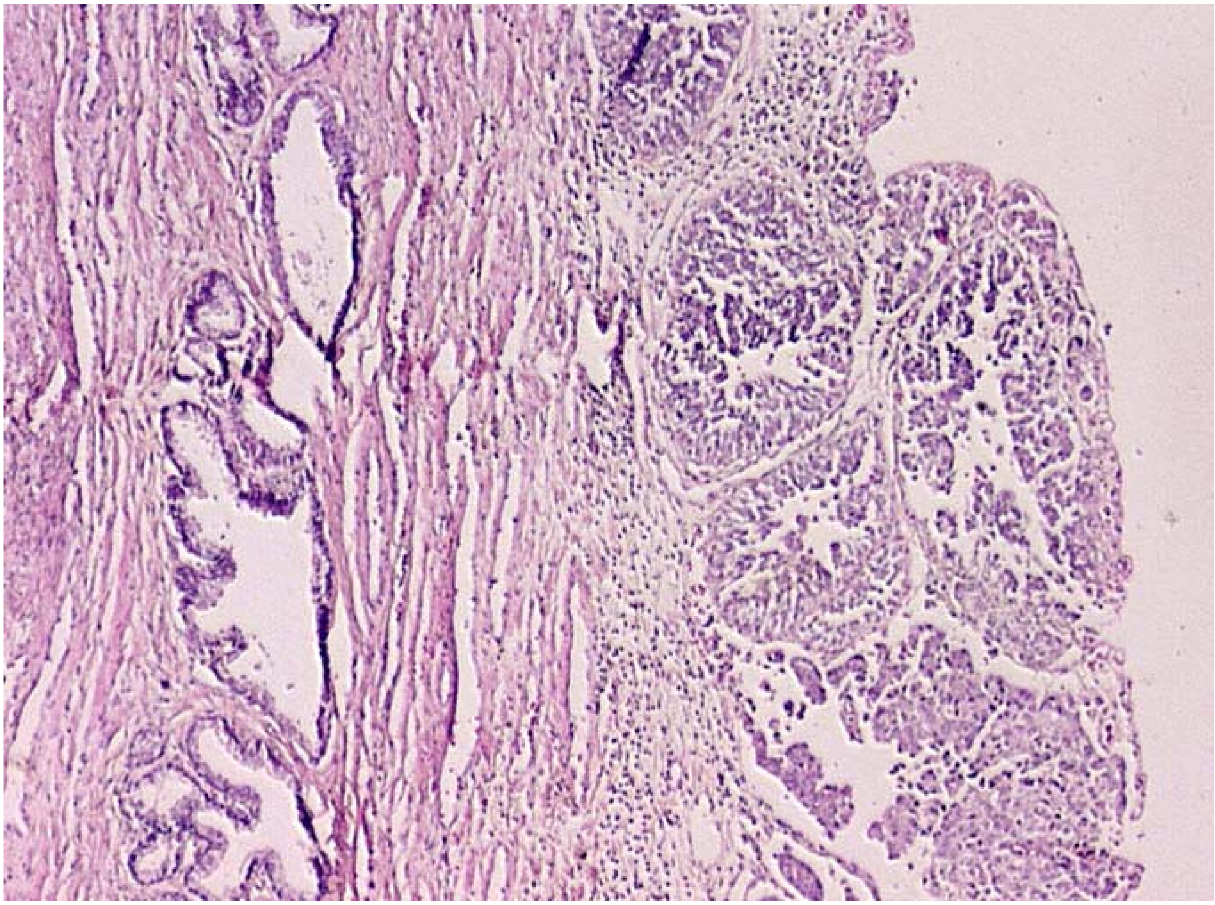

**FIGURE 1.** TCC of prostatic urethral epithelium (Hematoxylin-Eosin Stain, 40×).

Recently, Mungan et al.[13] investigated the factors affecting superficial involvement of the PU in patients with superficial bladder TCC. They evaluated the impact of pathological stage, grade, tumor multiplicity, and presence of CIS on mucosal PU involvement in a series of 340 male patients with the diagnosis of primary superficial bladder TCC. In univariate analysis, although tumor stage, grade, and multiplicity were found to be predicting factors, multivariate analysis revealed that only the bladder tumor multiplicity was found to be an independent predictor for superficial PU involvement by TCC. They demonstrated that the risk of superficial PU involvement with multiple tumors was 16 times higher than the risk of the tumor stage and grade[13]. They suggested that patients, particularly those with multiple superficial TCC of the bladder, seem to have an additional increased risk of superficial PU involvement.

Several studies have reported that the PU is a common site of recurrence after the use of BCG for bladder TCC[20,21]. Therefore, close surveillance of the PU in conservatively treated patients by cystoscopy and PU biopsy is important.

## DIAGNOSIS

Cystoscopy is the gold standard for detecting urothelial pathologies. Although preoperative cystoscopy was suggested to have a 83% sensitivity and 95% specifity for correctly diagnosing prostatic urethral TCC[22], TCC and even CIS might be present in the normal-appearing PU as isolated tumors[13]. Cystoscopic appearance of CIS is described as an erythematous, patchy, slightly raised, velvety lesion that has the potential for progression[14]. Particularly in patients with an enlarged, friable, and vascular prostate, it might be much more difficult to differentiate prostatic urethral CIS. Therefore, prostatic urethral sampling may be the best method for detecting prostatic urethral involvement by TCC and CIS.

Currently, controversy exists regarding the optimal technique to identify prostatic involvement by TCC. TUR biopsy or a cold-cup biopsy of the PU and prostate has been recommended in patients with bladder carcinoma, particularly those at high risk[13,23,24,25]. It was also suggested that prostatic urethral involvement might be present in patients with a positive urinary cytology following a complete response to intravesical therapy for bladder cancer[26]. Therefore, TUR biopsies of the prostate between the 5 and 7 o'clock positions along the entire length of the PU was recommended in order to detect prostatic urethral TCC in patients who have positive urinary cytology after complete TUR of bladder tumors or intravesical therapy[27]. Other less-invasive techniques might underestimate the extent of disease in these patients. Sakamoto et al.[7] suggested that TUR biopsy at the 5 and/or 7 o'clock position of the verumontanum improves the detection of prostatic duct and acini involvement by TCC in bladder cancer patients.

In one study, the significance of cystoscopically normal–appearing PU biopsies in patients who underwent cystectomy for invasive bladder cancer was investigated[28]. In this series, 40 men with cystoscopically normal prostatic fossa had negative prostatic biopsies and had orthotopic neobladder substitution. However, TCC involving the prostate was detected histopathologically in 3 out of these 40 patients after the pathological examination was performed. They concluded that TUR biopsy of the PU before cystectomy in patients with cystoscopically normal–appearing PU has limited value. Similarly, in another study, the specificity, sensitivity, and positive predictive value of TUR biopsies of the PU for predicting the degree of involvement at cystectomy was low[29]. Matzkin et al.[18] suggested that demonstration of stromal invasion is not also present on TUR biopses of the prostate, showing ductal involvement is difficult.

It is claimed that patients with BCG-refractory TCC of the bladder with a poor surgical risk for cystectomy may benefit from continued intravesical therapy without a significant risk of progression[30]. Very close follow-up in order to detect bladder recurrences, as well as prostatic urethral and upper urinary tract involvement, is necessary. The tumor in the PU at endoscopic staging was found to be the only factor associated with understaging and shorter survival in patients who underwent radical cystectomy for high-risk superficial bladder cancer after BCG failure[31].

## MANAGEMENT

The management of superficial urothelial cancer in the PU is unclear. Currently, there is increasing recognition of the value of conservative treatment options with intravesical agents when there is superficial involvement of the PU[23,32,33]. If TCC involves particularly the mucosa of the PU rather than the ducts or acinar structures, superficial tumor can respond to intravesical agents.

Chemotherapeutic agents like Mitomycin C, Adriamycin, Epirubicin, and BCG have been used as intravesical agents in the conservative management of superficial involvement of the PU by TCC. It was suggested that this therapy seems to have a prophylactic effect on tumor reccurrence in the prostate[23,32,33,34,35,36,37]. Among these agents, intravesical BCG seems to be an effective treatment alternative in the management of superficial mucosal PU involvement (Table 1). Because the intravesical agents only contact with the prostatic urothelium during instillation and voiding, the mechanism of action of their effects in treating prostatic TCC is not clear. However, detection of granuloma formation in the post-treatment biopsies of the prostate and occurrence of granulomatous prostatitis after intravesical BCG administration support the effective penetration of BCG in the PU and prostate[23,38]. Moreover, the detection of elevated interleukin 2 and interferon levels after BCG instillation suggests that BCG induces these cytokines, which have antitumor activity[39].

**TABLE 1**
**Complete Response (CR) Rates of Patients with Superficial Prostatic Urethral Involvement by TCC Who were Treated Conservatively by Intravesical Agents**

| Agent | Reference | CR (%) |
|---|---|---|
| Mitomycin-C or Adriamycin | Solsona et al.[37] | 37 |
| Epirubicin | Canda et al.[32] | 29 |
| BCG | Bretton et al.[35] | 48 |
| | Canda et al.[32] | 58 |
| | Hillyard et al.[23] | 75 |
| | Schellhammer et al.[34] | 70 |
| | Palou et al.[33] | 78 |
| | Orihuela et al.[36] | 87 |

## Low-Grade Superficial Urothelial Cancer in the PU

Patients with low-grade superficial involvement of the PU by TCC can be managed conservatively by TUR and chemo/immunotherapy, particularly with BCG[32]. In case of recurrence as low-grade tumors, another TUR and intravesical local treatment with BCG seems to be successful. Meticulous follow-up by cystoscopy and PU biopsy at 3-month intervals, particularly in intermediate and high-risk patients who respond to intravesical BCG therapy and in whom cystectomy is appropriate, can detect persistent tumor, recurrences, or progression[32].

## CIS and High-Grade Uurothelial Cancer in the PU

The success rates in treating high-grade tumors and CIS of the PU by BCG range from 70–100% in the prostate and from 47–72% considering bladder and prostate[14,23,33,34,35]. It is more difficult to treat prostatic CIS or high-grade superficial involvement of the PU by TCC. Palou et al.[33] reported complete response in 14 of 18 patients with CIS of the PU who were treated with intravesical BCG and 3 patients progressed. Successful management of CIS of the prostate by intravesical BCG has also been supported by other authors[23,35].

In a study of 50 patients with prostatic TCC without invasive bladder carcinoma, the 5-year disease-specific survival rate was 100% (n = 19) in patients with CIS of the PU and prostatic ducts and acini, whereas it was 45% (n = 21) in patients with TCC with stromal invasion[40].

Currently, it is well established that mucosal prostatic urethral involvement by urothelial cancer can be treated effectively with intravesical BCG administration, which is considered as the most effective treatment and prophylaxis for superficial TCC including CIS reducing tumor recurrence, disease progression, and mortality[41]. However, in those patients who fail conservative intravesical treatment with BCG, progression occurs more often in the bladder (40–60%) than in the prostate (20–30%)[42]. Therefore, close surveillance of the urinary tract including the PU in these patients is recommended.

## Prostatic Ductal Involvement by Urothelial Cancer

The management of prostatic ductal involvement by TCC is also debatable. Although some authors recommend a trial of BCG and meticulous follow-up by prostate biopsies in patients with minimal ductal involvement[43], others suggest radical cystoprostatectomy[44].

Bretton et al.[35] reported an 87% complete response rate for prostatic urethral TCC after the treatment with intravesical BCG administration, but only 4 of 23 patients had ductal involvement and none had stromal invasion. Schellhammer et al.[34] stated that TCC involving prostatic urothelium or periurethral ducts is not a contraindication to a trial of intravesical BCG. Others suggest that minimal ductal involvement by TCC does not preclude a trial of BCG and recommended careful follow-up and prostate biopsies[43].

Since prostatic ductal involvement has the potential for invasion, when invasion occurs, radical cystoprostatectomy alone may not be sufficient because of the high risk of metastatic disease. Thus, adjuvant or neoadjuvant chemotherapy may be needed[44].

Martinez-Pineiro et al.[45] compared the efficacy of one-third of the dose vs. standard dose intravesical BCG administration in decreasing the risk of recurrence and progression after TUR in a series of 155 patients with superficial high-risk (stages T1G3 and CIS) bladder TCC. Four patients (6.1%) with CIS were detected to have local extension into the PU and ducts, including 3 (8.3%) treated with the standard dose and 1 (3.4%) treated with the decreased dose BCG. They concluded that threefold-decreased dose and standard dose intravesical BCG are similarly effective in preventing progression in patients with high-risk stages (T1G3 and CIS) of superficial bladder carcinoma, with one-third of the dose BCG having significantly less toxicity[45].

In another study, the degree of TCC extension into the prostate and survival in 76 male patients who underwent radical cystectomy were investigated[12]. The authors detected similar survival rates in patients with prostatic urethral and ductal involvement by TCC without stromal invasion. It is suggested that survival is related with the stage of the primary bladder TCC.

In conclusion, superficial involvement of the PU by TCC is not a rare entity and is detected more commonly in bladder cancer patients with high-grade, multifocal tumors and with CIS. Although no prospective study currently exists assessing the value of prostatic urethral sampling in this patient group, taking biopsies from the PU particularly in this high-risk group might be important. In general, it is easy to identify prostatic urethral TCC by cystoscopy, however, it is important to keep in mind that these tumors may be present in the normal-appearing PU. Currently, a trial of intravesical BCG as a conservative treatment option is recommended in patients with superficial involvement of the PU by TCC with close follow-up by cystoscopy and PU biopsy.
